# Synthesis and Structural Characterization of a Metal Cluster and a Coordination Polymer Based on the [Mn_6_(*μ*_4_-O)_2_]^10+^ Unit

**DOI:** 10.1155/2010/367128

**Published:** 2010-06-07

**Authors:** Eleni E. Moushi, Anastasios J. Tasiopoulos, Manolis J. Manos

**Affiliations:** Department of Chemistry, University of Cyprus, 1678 Nicosia, Cyprus

## Abstract

A new 1-D coordination polymer {[Mn_6_O_2_(O_2_CMe)_10_(H_2_O)_4_]·2.5H_2_O}_*∞*_ (**1**·2.5H_2_O)_*∞*_ and the cluster [Mn_6_O_2_(O_2_(O_2_CPh)_10_ (py)_2_(MeCN)(H_2_O)]·2MeCN (**2**·2MeCN) are reported. Both compounds were synthesized by room temperature reactions of [Mn_3_(*μ*_3_-O)(O_2_CR)_6_(L)_2_(L′)] (R = Me, L = L′ = py, (**1**·2.5H_2_O)_*∞*_; R = Ph, L = py, L′ = H_2_O, **2**·2MeCN) in the presence of 3-hydroxymethylpyridine (3hmpH) in acetonitrile. The structures of these complexes are based on hexanuclear mixed-valent manganese carboxylate clusters containing the [Mn_4_^II^Mn_2_^III^(*μ*_4_-O)_2_]^10+^ structural core. (**1**·2.5H_2_O)_*∞*_ consists of zigzag chain polymers constructed from [Mn_6_O_2_(O_2_CMe)_10_(H_2_O)_4_] repeating units linked through acetate ligands, whereas **2**·2MeCN comprises a discrete Mn_6_-benzoate cluster.

## 1. Introduction

The synthesis of Mn clusters has attracted significant interest due to their relevance to many areas including molecular magnetism, catalysis, and bioinorganic chemistry [1, 2]. In the bioinorganic area, extensive work has been carried out to model the structure and catalytic activity of a tetranuclear Mn cluster, which is present in the water oxidizing centre (WOC) of Photosystem II [3–7]. As a result, a number of oligonuclear high oxidation state Mn-carboxylate clusters have been prepared [3, 5], some of which have been studied for their ability to oxidize H_2_O to molecular O_2_ [3, 6, 7]. Furthermore, considerable effort has been expended in order to prepare structural and reactivity models of other Mn-containing enzymes, such as Mn catalases. These studies have resulted in a number of oligonuclear Mn complexes with oxo/alkoxo/hydroxo or carboxylate bridges, some of which have proven to be very efficient catalytic scavengers of H_2_O_2_ [8]. The synthesis of oligonuclear Mn model compounds often involves preformed Mn carboxylate clusters and coordination polymers as starting materials, with the most popular ones being complexes based on the [Mn_3_O]^6+/7+^ and the [Mn_6_O_2_]^10+^ units [3, 9–11]. Since the various characteristics of the starting materials including their structural core, carboxylate bridges, and terminal ligation have a significant influence on the identity of the reaction product, there is always a need for new additions in the list of known metal precursor compounds.

Herein, we report the syntheses and the crystal structures of the 1D coordination polymer {[Mn_6_O_2_(O_2_CMe)_10_(H_2_O)_4_]·2.5H_2_O}_*∞*_ (**1**·2.5H_2_O)_∞_ and the discrete cluster [Mn_6_O_2_(O_2_CPh)_10_(py)_2_(MeCN)(H_2_O)]·2MeCN (**2**·2MeCN), which both contain the [Mn_4_^II^Mn_2_^III^(*μ*_4_-O)_2_]^10+^ structural core. Compound **2**·2MeCN is a new addition in the family of structurally-characterized Mn_6_-benzoate clusters [12, 13], whereas (**1**·2.5H_2_O)_∞_ represents one of the few coordination polymers based on hexanuclear Mn clusters [14–17].

## 2. Experimental

### 2.1. Materials

All manipulations were performed under aerobic conditions using materials (reagent grade) and solvents as received; water was distilled in-house. [Mn_3_O(O_2_CMe)_6_(py)_3_]·py and [Mn_3_O(O_2_CPh)_6_(py)_2_(H_2_O)]·0.5CH_3_CN were prepared as described elsewhere [18]. 

### 2.2. Compound Preparation

{[Mn_6_O_2_(O_2_CMe)_10_· (H_2_O)_4_]·2.5H_2_O}_*∞*_ (**1**·2.5H_2_O)_∞_: [Mn_3_O(O_2_CMe)_6_(py)_3_]*·*py (0.2 g, 0.24 mmol) was dissolved in MeCN (10 mL), and then 3 hmpH (0.05 g, 0.46 mmol) was added to the dark brown solution. The resulting red-brown solution was left under magnetic stirring for ~50 minutes, filtered off, and the filtrate was left undisturbed at room temperature. After a few weeks, brown crystals of (**1**·2.5H_2_O)_∞_ suitable for X-ray crystallography were formed. The crystals were collected by filtration, washed with MeCN (10 mL), and Et_2_O (2 × 10 mL) and dried *in vacuo*. The yield was ~27% based on total Mn content. *Anal. Calc.* for C_20_H_43_Mn_6_O_28.5 _(**1**·2.5H_2_O)_∞_: C, 22.47; H, 4.05. Found: C 22.29; H 4.25%. IR data (KBr pellet, cm^−1^): $\tilde{v}$ = 3404 (m,br), 1582 (s), 1421 (s), 1371 (w), 1028 (m), 667 (s), 619 (s).

[Mn_6_O_2_(O_2_CPh)_10_(py)_2_(MeCN)(H_2_O)]·2MeCN (**2**·2MeCN): [Mn_3_O(O_2_CPh)_6_(py)_2_(H_2_O)]·0.5CH_3_CN (0.27 g, 0.24 mmol) was dissolved in MeCN (10 mL) and then, 3 hmpH (0.05 g, 0.46 mmol) was added to the dark brown solution. The resulting red-brown solution was left under magnetic stirring for ~45 minutes, filtered off and the filtrate was left undisturbed at room temperature. After a few weeks, brown crystals of (**2**·2MeCN) suitable for X-ray crystallography were formed. The crystals were collected by filtration, washed with MeCN (10 mL) and Et_2_O (2 × 10 mL) and dried *in vacuo*. The yield was ~20% based on total Mn content. *Anal. Calc.* for C_86_H_71_Mn_6_N_5_O_23 _(**2**·2MeCN): C, 55.17; H, 3.82; N, 3.74. Found: C 54.98; H 3.91; N, 3.53%. IR data (KBr pellet, cm^−1^): $\tilde{v}$ = 3398 (m,br), 1607 (s), 1570 (s), 1430 (s) 720 (s), 691 (m), 676 (m), 614 (m).

### 2.3. X-Ray Crystallography

Data were collected on an Oxford-Diffraction Xcalibur diffractometer, equipped with a CCD area detector and a graphite monochromator utilizing Mo-K*α* radiation (*λ* = 0.71073 Å). Suitable crystals were attached to glass fibers using paratone-N oil and transferred to a goniostat where they were cooled for data collection. Unit cell dimensions were determined and refined by using 4714 (3.14 ≤ *θ* ≤ 30.42°) and 23078 (3.07 ≤ *θ* ≤ 31.25°) reflections for (**1**·2.5H_2_O)_∞_ and **2**·2MeCN, respectively. Empirical absorption corrections (multiscan based on symmetry-related measurements) were applied using CrysAlis RED software [19]. The structures were solved by direct methods using SIR92 [20] and refined on *F* ^2^ using full-matrix least squares with SHELXL97 [21]. Software packages used: CrysAlis CCD [19] for data collection, CrysAlis RED [19] for cell refinement and data reduction, WINGX for geometric calculations [22], and DIAMOND [23] and MERCURY [24] for molecular graphics. The non-H atoms were treated anisotropically, whereas the aromatic and methyl-hydrogen atoms were placed in calculated, ideal positions and refined as riding on their respective carbon atoms. The H atoms of water molecules could not be located. Unit cell data and structure refinement details are listed in Table 1. 

### 2.4. Physical Measurements

Elemental analyses (C, H, N) were performed by the in-house facilities of the University of Cyprus, Chemistry Department. IR spectra were recorded on KBr pellets in the 4000–400 cm^−1^ range using a Shimadzu Prestige-21 spectrometer. 

## 3. Results and Discussion

### 3.1. Syntheses

The goal of the described research is the synthesis of multidimensional coordination polymers composed of polynuclear Mn carboxylate clusters with the use of hydroxymethyl-pyridine derivatives [e.g., 4-hydroxymethyl-pyridine (4hmpH), 3-hydroxymethyl-pyridine (3hmpH)] as bridging ligands. The initial result from these investigations was a new hexanuclear Mn complex [Mn_6_O_2_(O_2_CPh)_10_(4hmpH)_3_(MeCN)], which contains the [Mn_6_O_2_]^10+^ structural core and terminal 4 hmpH ligands [12]. This compound was prepared from a reaction of [Mn(O_2_CPh)_2_]·2H_2_O with 4hmpH in MeCN. Various modifications of this reaction system that were performed involved the use of preformed Mn clusters as precursor compounds together with 3hmpH. Thus, the reaction of [Mn_3_(*μ*_3_-O)(O_2_CR)_6_(L)_2_(L′)] (R = Me, L = L′ = py, (**1**·2.5H_2_O)_∞_;R = Ph, L = py, L′ = H_2_O, **2**·2MeCN) with 3hmpH in acetonitrile resulted in the isolation of compounds (**1**·2.5H_2_O)_∞_ and **2**·2MeCN, which however did not contain the 3hmpH ligands. The formation of (**1**)_∞_ and** 2 **is summarized in equations 1 and 2, respectively:

(1) $$\begin{array}{cc} & 2\text{n}\left[{\text{Mn}}_{3}\text{O}{\left({\text{O}}_{2}\text{CMe}\right)}_{6}{\left(\text{py}\right)}_{3}\right]+4{\text{nH}}_{2}\text{O}+2{\text{ne}}^{-}\\ & \;\to {\left[{\text{Mn}}_{6}{\text{O}}_{2}{\left({\text{O}}_{2}\text{CMe}\right)}_{10}{\left({\text{H}}_{2}\text{O}\right)}_{4}\right]}_{\text{n}}+2{{\text{nMeCO}}_{2}}^{-}+6\text{npy} \end{array}$$

(2) $$\begin{array}{cc} & 2\left[{\text{Mn}}_{3}\text{O}{\left({\text{O}}_{2}\text{CPh}\right)}_{6}{\left(\text{py}\right)}_{2}\left({\text{H}}_{2}\text{O}\right)\right]+\text{MeCN}+2{\text{e}}^{-}\\ & \;\;\to \left[{\text{Mn}}_{6}{\text{O}}_{2}{\left({\text{O}}_{2}\text{CPh}\right)}_{10}{\left(\text{py}\right)}_{2}\left(\text{MeCN}\right)\left({\text{H}}_{2}\text{O}\right)\right]\\ & \;\;\;+2{{\text{PhCO}}_{2}}^{-}+2\text{py}+{\text{H}}_{2}\text{O} \end{array}$$

As it will be discussed in detail below, the structures of (**1**·2.5H_2_O)_∞_ and **2**·2MeCN are very similar with one major difference between them being the fact that (**1**·2.5H_2_O)_∞_ is a coordination polymer, whereas **2**·2MeCN is a discrete metal cluster. A possible explanation for this is that the bulky PhCO_2_^−^ groups that are present in **2**·2MeCN prevent the polymerization of the Mn_6_ clusters, whereas in (**1**·2.5H_2_O)_∞ _there are only acetate ligands that are more flexible and thus can easily bridge Mn_6_ units leading to a polymeric species. We also note that the average oxidation state of the final products (2.33) of the two reactions is lower than that of the starting materials (2.66). Such a reduction could be explained assuming that a disproportionation reaction of the Mn_3_ starting materials takes place upon their dissolution in MeCN in the presence of 3hmpH. Then, the reduced species are aggregated to form (**1**·2.5H_2_O)_∞_ or **2**·2MeCN and the products with Mn ions in higher oxidation states remain in the solution. Similar reactions as those leading to the isolation of (**1**·2.5H_2_O)_∞ _or **2**·2MeCN were performed using 4hmpH or pyridine instead of 3hmpH in the reaction mixtures. These reactions resulted in the isolation of microcrystalline products that have not been completely characterized so far, but seem to be different than compounds (**1**·2.5H_2_O)_∞_ and** 2**·2MeCN (by comparisons of infrared spectra). Reactions were also carried out by us in the past, where no other reagent (e.g., pyridine or triethylamine) was used besides the [Mn_3_O(O_2_CMe)_6_(py)_3_] precursor compound and the solvent. In that case, an 1D coordination polymer based on Mn_3_-carboxylate cluster linked by Mn^2+^ ions was isolated [25]. Therefore, 3hmpH seems to play an important role in the formation of compounds (**1**·2.5H_2_O)_∞_ and **2**·2MeCN, since different compounds are isolated in the absence of 3hmpH. However, the exact role of 3 hmpH in the assembly of these compounds is yet unidentified.

### 3.2. Crystal Structures

The structure of the repeating unit of (**1**·2.5H_2_O)_∞_ is very similar to that of compound **2**·2MeCN (with the main differences between the two compounds being the terminal ligation and the type of carboxylate ligands) and thus, only the first one will be discussed in detail. Selected interatomic distances for (**1**·2.5H_2_O)_∞_ and **2**·2MeCN are given in Tables 2 and 3, respectively.

**Figure 1:**
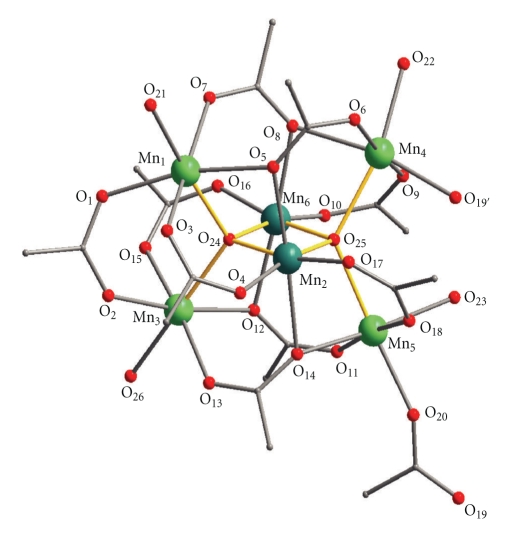
A partially labeled plot of the repeating unit of (**1**)_∞_. The yellow lines emphasize the [Mn_4_^II^Mn_2_^III^(*μ*_4_-O)_2_]^10+^ core. Color code: Mn^II^, green; Mn^III^, dark green; O, red; C, grey. H atoms are omitted for clarity.

Compound (**1**·2.5H_2_O)_∞_ crystallizes in the orthorhombic space group *Pbca*. Its repeating unit comprises the hexanuclear cluster [Mn_6_O_2_(O_2_CMe)_10_(H_2_O)_4_] (Figure 1) and totally 2.5H_2_O molecules of crystallization. Charge considerations, bond valence sum calculations (Table 4) and inspection of metric parameters indicate that the cluster is mixed-valent containing four Mn^II^ and two Mn^III^ ions. The [Mn_4_^II^Mn_2_^III^(*μ*_4_-O)_2_]^10+^ core of **1** has appeared several times in the literature as will be discussed in detail below and can be described as consisting of two edge-sharing (*μ*_4_-O)Mn_4_ tetrahedra. Such units are defined as anti-T1 tetrahedra (T1 is a structural unit having a cation at the center and four anions at the apices of the tetrahedron) [26]. The common edge of the two anti-T1 tetrahedra is formed by the two Mn^III^ ions, whereas the four Mn^II^ ions occupy the corners of the [Mn_4_^II^Mn_2_^III^(*μ*_4_-O)_2_]^10+^ core. The peripheral ligation of the Mn atoms is completed by 4 terminal H_2_O molecules (ligated to the four Mn^II^ atoms) and 10 acetate ligands.

**Figure 2:**
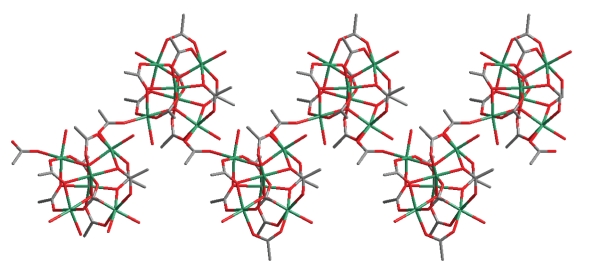
Wireframe representation of the zigzag chain of (**1**)_∞_ viewed along *a*-axis. Mn, green; O, red; C, grey. H atoms are omitted for clarity.

**Figure 3:**
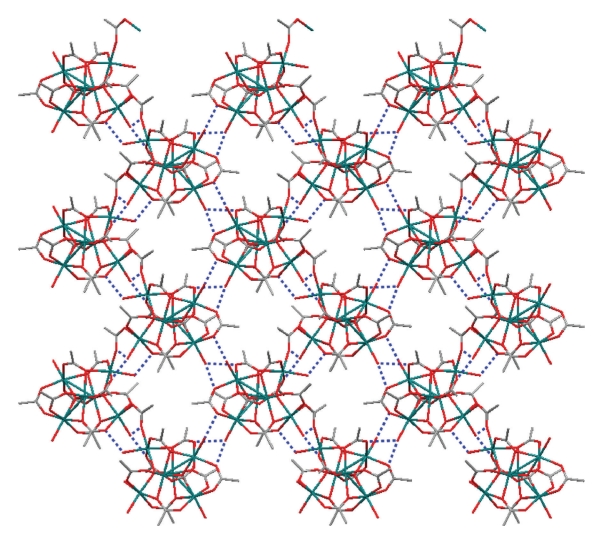
Wireframe representation of the layer formed by interchain hydrogen bonds in (**1**)_∞_. The hydrogen bonds are shown as dotted blue lines. Mn, green; O, red; C, grey. H atoms are omitted for clarity.

All Mn atoms are in distorted octahedral geometries. Five of the intra-cluster acetate groups are *μ*_2_ with each of their carboxylate oxygen atoms acting as terminal ligand for a Mn center. Four acetate ligands are coordinated in *η* ^1^ : *η* ^2^ : *μ*_3_ fashion. The remaining carboxylate ligand bridges two Mn^II^ atoms (Mn⋯Mn distance = 4.7914(2) Å) of adjacent Mn_6_ clusters, thus resulting in the formation of a zigzag chain structure (Figure 2). The chains are interacting through hydrogen bonds (O⋯O distances 2.7−2.9 Å) involving the coordinated water molecules and carboxylate O atoms. Thus, a two-dimensional hydrogen-bonded polymer with a 4-connected topology is formed (Figure 3). The hydrogen bonds involving the lattice water molecules cannot be identified with accuracy due to the positional disorder of these molecules and thus, are not discussed here. 

**Figure 4:**
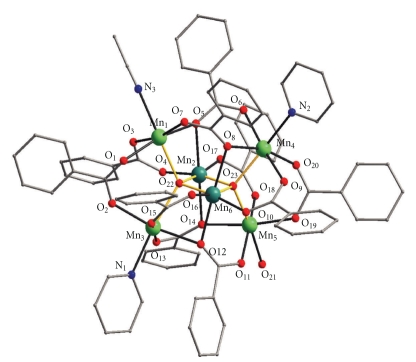
A partially labeled plot of **2**. The yellow lines emphasize the [Mn_4_^II^Mn_2_^III^(*μ*_4_-O)_2_]^10+^ core. Color code: Mn^II^, green; Mn^III^, dark green; O, red; N, blue; C, grey. H atoms are omitted for clarity.

A representation of the structure of **2**·2MeCN is given in Figure 4. The structure of **2**·2MeCN is very similar to that of (**1**·2.5H_2_O)_∞_ with the main differences between them being (i) the type of terminal ligands [4H_2_O for (**1**·2.5H_2_O)_∞_; 2 py, one MeCN and one H_2_O for **2**·2MeCN] (ii) the type of carboxylate groups (acetate for (**1**·2.5H_2_O)_∞_; benzoate for** 2**·2MeCN) and (iii) their dimensionality [(**1**·2.5H_2_O)_∞_ is a coordination polymer, whereas **2**·2MeCN is a discrete metal cluster]. Regarding point (iii) we note that examination of the packing of **2**·2MeCN revealed the existence of intermolecular hydrogen bonding interactions (O⋯O distances 2.792(4) and 2.809(4) Å) involving the terminal H_2_O molecule and two O_benzoate_ atoms of two neighboring Mn_6_ molecules resulting in the formation of a dimeric (**2**·2MeCN)_2_ aggregate.

The Mn_6_ unit that appears in (**1**·2.5H_2_O)_∞_ and **2**·2MeCN, that is, the cluster [Mn_6_O_2_(O_2_CR)_10_(L)_2_(L′) (L′′)] (R = Me, L = L′ = L′′ = H_2_O, **1**; R = Ph, L = py, L′ = H_2_O, L′′ = MeCN, **2**), has a structural motif found in several hexanuclear Mn clusters and coordination polymers [9, 12–16]. For example, we have recently reported the discrete cluster [Mn_6_O_2_(O_2_CPh)_10_(4hmpH)_3_(MeCN)] containing the [Mn_4_^II^Mn_2_^III^(*μ*_4_-O)_2_]^10+^ core and also three terminal 4hmpH groups linked through their N_pyridine_ atom and a MeCN molecule [12]. In addition, compound (**1**·2.5H_2_O)_∞_ is closely related to compound {[Mn_6_O_2_(O_2_CEt)_10_(H_2_O)_4_]·2EtCO_2_H}_*∞*_ (**3**·2EtCO_2_H)_∞_, recently published [14]. The main structural differences between them lie in the type of carboxylate ligands in these compounds, being acetate groups in (**1**·2.5H_2_O)_∞_ and propionate ligands in (**3**·2EtCO_2_H)_∞_ and also in the type of the crystallization solvent molecules. Other related examples to (**1**·2.5H_2_O)_∞_ comprise the chain polymers [Mn_6_O_2_(O_2_CCMe_3_)_10_(thf)_2_(NIT-Me)][Mn_6_O_2_(O_2_CCMe_3_)_10_(thf)(CH_2_Cl_2_)(NIT-Me)] (thf = tetrahydrofuran, NIT-Me = 4,5-dihydro-1*H*-imidazolyl-3-oxide-1-oxyl) [15] and [Mn_6_O_2_(O_2_CCMe_3_)_10_(HO_2_CCMe_3_)_2_(bpy)] (bpy = 4,4′-bipyridine) [16].

## 4. Conclusions

We reported the syntheses and the crystal structures of compounds (**1**·2.5H_2_O)_∞_ and **2**·2MeCN, which are based on the well-known [Mn_6_(*μ*_4_-O)_2_]^10+^ structural core. Both compounds were prepared serendipitously in our attempt to prepare polymeric species consisting of polynuclear Mn clusters linked through 3 hmpH. Compound (**1**·2.5H_2_O)_∞ _features a zigzag chain structure formed by [Mn_6_(*μ*_4_-O)_2_(O_2_CMe)_10_(H_2_O)_4_] clusters linked via bridging acetate ligands. This compound joins a family of coordination polymers based on the [Mn_6_(*μ*_4_-O)_2_]^10+^ unit, which numbers only a few members. Furthermore, compound **2**·2MeCN represents a new addition in the growing family of Mn_6_-benzoate clusters. Further work may involve replacement of the terminal solvent molecules in (**1**·2.5H_2_O)_∞ _or **2**·2MeCN by various bridging polytopic ligands, in order to isolate higher dimensionality (2D, 3D) polymers. Multidimensional coordination polymers consisting of oligonuclear Mn clusters would be potential candidates for various applications including gas storage and catalysis.

## Figures and Tables

**Table 1:** Crystallographic data for complexes (**1**·2.5H_2_O)_∞_ and (**2**·2MeCN).

	**1**	**2**
Formula^a^	C_40_H_86_Mn_12_O_57_	C_86_H_71_Mn_6_N_5_O_23_
*M*_*w*_	2138.33	1872.13
Crystal System	Orthorhombic	Triclinic
Space group	P b c a	Pī
*a*/Å	13.615(2)	14.4690(8)
*b*/Å	21.274(3)	15.8172(7)
*c*/Å	30.459(4)	18.636(2)
*α*/^*ο*^	90	83.861(4)
*β*/^*ο*^	90	86.750(4)
*γ*/^*ο*^	90	83.463(4)
*V*/Å^3^	8822(2)	4208.8(4)
*Z *	4	2
*T*/K	100(2)	100(2)
*λ* ^b^, Å	0.71073	0.71073
*D*_*c*_, g/cm^−3 a^	1.610	1.477
*μ* (Mo-K*α*)/mm^−1^	1.750	0.950
Reflections collected/unique(*R*_int _)	38727/7727(0.1214)	46797/11574(0.0493)
Obs. refl. [I > 2*σ*(I)].	3263	8550
*R*1%^c^	0.0474	0.0656
*w* *R*2^d^	0.0931	0.1608
Goodness of fit on *F* ^2^	0.807	0.974
Δ*ρ* max/min/*e* Å^−3^	0.891/−0.409	1.434/−1.333

^a^Including solvent molecules and all hydrogen atoms (even the H atoms of H_2_O). ^b^Graphite monochromator. ^c^ *R*1 = Σ|*F*_*ο*_| − |*F*_*c*_|/Σ|*F*_*ο*_|. ^d^ *w* *R*2 = [Σ[*w*(*F*_*o*_^2^−*F*_*c*_^2^)^2^]/Σ[*w* *F*_*o*_^2^)^2^]]^1/2^, *w* = 1/[*σ*2(*F*_*ο*_^2^) + (*m*·*p*)^2^ + *n* · *p*], *p* = [max (*F*_*o*_^2^, 0) + 2*F*_*c*_^2^]/3, and *m* and *n* are constants.

**Table 2:** Selected interatomic distances (Å) for complex (**1**·2.5H_2_O)_∞_.

Bond distances (Å)			
Mn1⋯Mn2	3.138(2)	Mn3–O26	2.186(4)
Mn2⋯Mn6	2.798(2)	Mn3–O24	2.195(4)
Mn2⋯Mn5	3.164(2)	Mn3–O12	2.201(4)
Mn3⋯Mn6	3.131(2)	Mn4–O19	2.138(5)
Mn4⋯Mn6	3.187(2)	Mn4–O22	2.160(5)
Mn1–O7	2.130(5)	Mn4–O6	2.173(5)
Mn1–O24	2.162(4)	Mn4–O9	2.191(5)
Mn1–O3	2.164(5)	Mn4–O25	2.240(4)
Mn1–O21	2.172(4)	Mn4–O8	2.313(5)
Mn1–O1	2.176(4)	Mn5–O20	2.125(5)
Mn1–O5	2.293(5)	Mn5–O11	2.142(5)
Mn2–O25	1.883(4)	Mn5–O18	2.193(5)
Mn2–O24	1.891(4)	Mn5–O14	2.193(4)
Mn2–O4	1.943(4)	Mn5–O23	2.207(4)
Mn2–O17	1.962(5)	Mn5–O25	2.265(4)
Mn2–O5	2.229(4)	Mn6–O24	1.892(4)
Mn2–O14	2.257(4)	Mn6–O25	1.894(5)
Mn3–O2	2.143(4)	Mn6–O10	1.931(4)
Mn3–O15	2.155(5)	Mn6–O16	1.973(5)
Mn3–O13	2.163(5)	Mn6–O8	2.202(4)
		Mn6–O12	2.232(4)

**Table 3:** Selected interatomic distances (Å) for complex (**2**·2MeCN).

Bond Distances (Å)			
Mn1⋯Mn2	3.133(2)	Mn3–O22	2.206(3)
Mn2⋯Mn6	2.8134(9)	Mn3–N1	2.263(4)
Mn2⋯Mn5	3.130(2)	Mn3–O12	2.317(3)
Mn3⋯Mn6	3.162(2)	Mn4–O6	2.124(3)
Mn4⋯Mn6	3.167(2)	Mn4–O20	2.131(3)
Mn1–O1	2.121(3)	Mn4–O9	2.154(3)
Mn1–O7	2.121(3)	Mn4–O23	2.186(3)
Mn1–O3	2.145(3)	Mn4–N2	2.287(4)
Mn1–O22	2.190(3)	Mn4–O8	2.302(3)
Mn1–N3	2.250(5)	Mn5–O19	2.155(3)
Mn1–O5	2.281(3)	Mn5–O18	2.158(3)
Mn2–O22	1.872(3)	Mn5–O23	2.176(3)
Mn2–O23	1.894(3)	Mn5–O11	2.197(3)
Mn2–O17	1.936(3)	Mn5–O21	2.226(3)
Mn2–O4	1.972(4)	Mn5–O14	2.237(4)
Mn2–O5	2.239(3)	Mn6–O22	1.889(3)
Mn2–O14	2.241(3)	Mn6–O23	1.893(3)
Mn3–O2	2.127(4)	Mn6–O10	1.951(4)
Mn3–O13	2.146(3)	Mn6–O16	1.954(3)
Mn3–O15	2.151(3)	Mn6–O12	2.202(3)
		Mn6–O8	2.232(3)

**Table 4:** Bond valence sum (BVS)^a,b^ calculations for complexes (**1**·2.5H_2_O)_∞_ and (**2**·2MeCN)

	Complex** 1**			Complex** 2**		
	Mn^II^	Mn^III^	Mn^IV^	Mn^II^	Mn^III^	Mn^IV^
Mn1	**1.96**	1.79	1.88	**2.03**	1.87	1.94
Mn2	3.20	**2.92**	3.07	3.21	**2.94**	3.08
Mn3	**1.99**	1.82	1.91	**1.94**	1.79	1.85
Mn4	**1.86**	1.70	1.79	**1.96**	1.81	1.87
Mn5	**1.93**	1.76	1.85	**1.90**	1.74	1.83
Mn6	3.21	**2.94**	3.09	3.22	**2.94**	3.09

^a^The bold value is the one closest to the charge for which it was calculated. ^b^The oxidation state is the nearest whole number to the bold value.
